# The learning environment and resident burnout: a national study

**DOI:** 10.1007/s40037-018-0405-1

**Published:** 2018-02-23

**Authors:** Stefan N. van Vendeloo, David J. Prins, Cees C. P. M. Verheyen, Jelle T. Prins, Fleur van den Heijkant, Frank M. M. A. van der Heijden, Paul L. P. Brand

**Affiliations:** 1 Department of Orthopedic Surgery and Traumatology, Isala Hospital, Zwolle, The Netherlands; 2 Department of Pulmonology, Medical Center Leeuwarden, Leeuwarden, The Netherlands; 3 MCL Academy, Medical Center Leeuwarden, Leeuwarden, The Netherlands; 4 Department of Urology, Maastricht University Medical Centre, Maastricht, The Netherlands; 5 Vincent van Gogh Institute for Psychiatry, Venray, The Netherlands; 6 Department of Pediatrics, Isala Hospital, Zwolle, The Netherlands; 7 Postgraduate School of Medicine, University Medical Center Groningen, Groningen, The Netherlands

**Keywords:** Learning environment, Burnout, Residency, Postgraduate medical education

## Abstract

**Introduction:**

Concerns exist about the negative impact of burnout on the professional and personal lives of residents. It is suggested that the origins of burnout among residents are rooted in the learning environment. We aimed to evaluate the association between the learning environment and burnout in a national sample of Dutch residents.

**Methods:**

We conducted a cross-sectional online survey among all Dutch residents in September 2015. We measured the learning environment using the three domain scores on content, organization, and atmosphere from the Scan of Postgraduate Educational Environment Domains (SPEED) and burnout using the Dutch version of the Maslach Burnout Inventory (UBOS-C).

**Results:**

Of 1,231 responding residents (33 specialties), 185 (15.0%) met criteria for burnout. After adjusting for demographic (age, gender and marital status) and work-related factors (year of training, type of teaching hospital and type of specialty), we found a consistent inverse association between SPEED scores and the risk of burnout (aOR 0.54, 95% CI 0.46 to 0.62, *p* < 0.001).

**Discussion:**

We found a strong and consistent inverse association between the perceived quality of the learning environment and burnout among residents. This suggests that the learning environment is of key importance in preventing resident burnout.

## What this paper adds

Recent insights indicate that burnout is not a problem of the individual but of the environment in which he/she works. However, the role the learning environment plays in the development of resident burnout is poorly studied. In this paper we describe a consistent association between the quality of the learning environment as perceived by residents and the development of burnout.

## Introduction

The virtues of medical residency are offset by high educational demands, long working hours, lack of autonomy, a high level of work-home interference and a lack of reciprocity in professional relationships. These factors may have detrimental effects on the mental health of residents and a substantial proportion of residents experience symptoms of burnout [1–3]. Three dimensions define the multifaceted syndrome of burnout: emotional exhaustion, depersonalization, and reduced personal accomplishment [4]. Burnout has both professional and personal implications. Residents with burnout are more likely to deliver suboptimal patient care and are at greater risk of making medical errors [5, 6]. In addition, residents with burnout show increased rates of substance abuse, alcohol consumption and suicidal thoughts [7, 8].

These days physician burnout is viewed as being rooted in issues related to the working environment and organizational culture, instead of being an individual problem [9, 10]. When it concerns resident doctors, this working environment resembles the learning environment [11], which is a construct that includes formal and informal aspects of the training program, organizational aspects within the teaching hospital [12] as well as the overall atmosphere [13]. The learning environment is thought to play a key role in the development of residents towards independent practice [14] and it has been postulated to be an important contributor to burnout [2]. A previous content analysis of instruments that assess the learning environment showed that the majority of the items of these instruments relate to a theoretical framework that characterizes the learning environment in three broad domains: the *content* of the program, the interpersonal aspects and *atmosphere* of the program, and the structure and *organization* of the program [15].

The learning environment plays a vital role in the development of burnout among medical students [16] and in a previous study we found that a better learning environment was associated with fewer symptoms of burnout and a better quality of life in orthopaedic residents [17]. However, it is unknown whether the association found in medical students can be translated to residents and whether an association between the learning environment and burnout exists across specialties. As work conditions across specialties and the personalities of these residents might differ, it is likely that the previous results we found among orthopaedic residents are not generalizable to other specialties.

The aim of the present study was therefore to examine the relationship between the perceived quality of the learning environment and the development of resident burnout in a large national sample of Dutch residents from all specialties. We hypothesized that the inverse association between the perceived quality of the learning environment and the development of burnout is present across specialties. Secondarily, we aimed to determine the effect size of the learning environment by controlling for other demographic and occupational predictors of resident burnout.

## Methods

### Settings and participants

In September 2015, a total of 7,141 residents were registered by the national Dutch Registration Commission of Medical Specialties (Registratiecommissie Geneeskundige Specialismen, RGS) as being enrolled in one of the postgraduate medical training programs in the Netherlands. Of these 7,141, 2,596 (36.4%) were members of the Dutch Junior Doctor Association. All these 2,596 members received an invitation by email on 21 September 2015 to participate in our study and complete an online self-report survey. Members of the association were encouraged to share the link for the survey with their fellow non-member residents.

Following the Netherlands Society of Medical Education guidelines for educational research and in accordance with the Declaration of Helsinki, anonymity was guaranteed, participation was voluntary, and informed consent was obtained.

### Survey

We used an abbreviated version of the Scan of Postgraduate Educational Environment Domains (SPEED) [15] to measure the perceived quality of the learning environment. We chose the SPEED because it is a validated, concise and theoretically well-founded instrument to evaluate the quality of the learning environment in the Dutch postgraduate medical education context [15]. We used the three items that provide an overall numerical rating of the quality of each domain (content, atmosphere and organization) of the learning environment. Respondents assessed these items on a scale ranging from ‘very poor’ (1) to ‘excellent’ (10). Means were calculated for each domain and these mean domain scores were used to calculate an overall mean SPEED score, which provides an overall rating for the learning environment.

We used the validated Dutch version (UBOS-C) [18] of the Maslach Burnout Inventory (MBI) [4] to measure burnout. It consists of 20 items covering the three domains of burnout: emotional exhaustion (8 items), depersonalization (5 items) and personal accomplishment (7 items). Items were scored on a 7-point Likert scale ranging from ‘never’ (0) to ‘always’ (6). Mean scores were calculated for each domain. We used cut-off scores for burnout based on a reference group of 10,552 Dutch healthcare employees [18]. A resident was diagnosed with burnout if there was either a mean score ≥2.50 on emotional exhaustion and ≥1.80 (men) or ≥1.60 (women) on depersonalization, or a mean score ≥2.50 on emotional exhaustion and a mean score of ≤3.70 on personal accomplishment [18].

Respondents provided information on: gender, age, marital status, type of medical specialty, year of postgraduate training, clinical setting (academic centre/affiliated teaching hospital), number of hours stated in their employment contracts and true number of hours worked.

### Data analysis

All analyses were done using SPSS version 17 (SPSS Inc., Chicago, Illinois, US). Standard descriptive summary statistics were used to characterize the sample. The representativeness of our study population was assessed by comparing age, gender, and type of specialty of respondents to those of all 7,141 residents enrolled in a postgraduate medical educational program at the time of study (RGS data). Student’s t tests were computed to compare mean SPEED scores (learning environment) between residents with and without burnout. Multivariate logistic regression analysis was conducted to evaluate the association between learning environment (SPEED scores) and resident burnout, adjusted for potential predictors of burnout. In this logistic regression model, we adjusted for demographic (age, gender and marital status) and work-related factors (year of training, type of teaching hospital and type of specialty). All tests used were two-tailed and *p*-values <0.05 were considered significant.

## Results

A total of 1,231 residents from 33 different specialties completed the survey, representing 17.2% of the total number of residents enrolled in postgraduate medical educational programs at the time the study was conducted. Because of our sampling strategy, an exact response rate could not be calculated. Our study sample was representative of the root population of all Dutch residents in terms of age and type of specialty; women were overrepresented in our study sample (73.6%) compared with the national root population of all residents (64.2%) (*p* < 0.01). Tab. 1 shows the demographic and occupational characteristics of the responding residents. The mean score (SD) on the three SPEED domains was 7.33 (1.01) (Tab. 1). A total of 185 residents (15.0%) fulfilled the criteria for burnout (Tab. 1), of which 47 (25.4%, or 3.8% of the study population) suffered from severe burnout.

**Table 1:** Demographic and occupational characteristics of participating residents

	Number (%)	Mean score (SD)
*Gender*		
Male	325 (26.4)	
Female	906 (73.6)	
*Age, years; median (range)*	32 (26–40)	
*Marital status*		
Married or cohabiting	960 (78.0)	
Single	253 (20.6)	
Other (e. g. with parents)	18 (1.4)	
*Years in training*		
1	218 (17.7)	
2	252 (20.5)	
3	275 (22.3)	
4	218 (17.7)	
5	179 (14.5)	
6	73 (5.9)	
7	5 (0.4)	
Just finished training	11 (0.9)	
*Burned out*	185 (15.0)	
High score emotional exhaustion	314 (25.5)	
High score depersonalization	292 (23.7)	
Low score personal accomplishment	163 (13.2)	
*SPEED*		7.33 (1.01)
Content		7.54 (1.07)
Atmosphere		7.59 (1.25)
Organisation		6.86 (1.33)

**Fig. 1:**
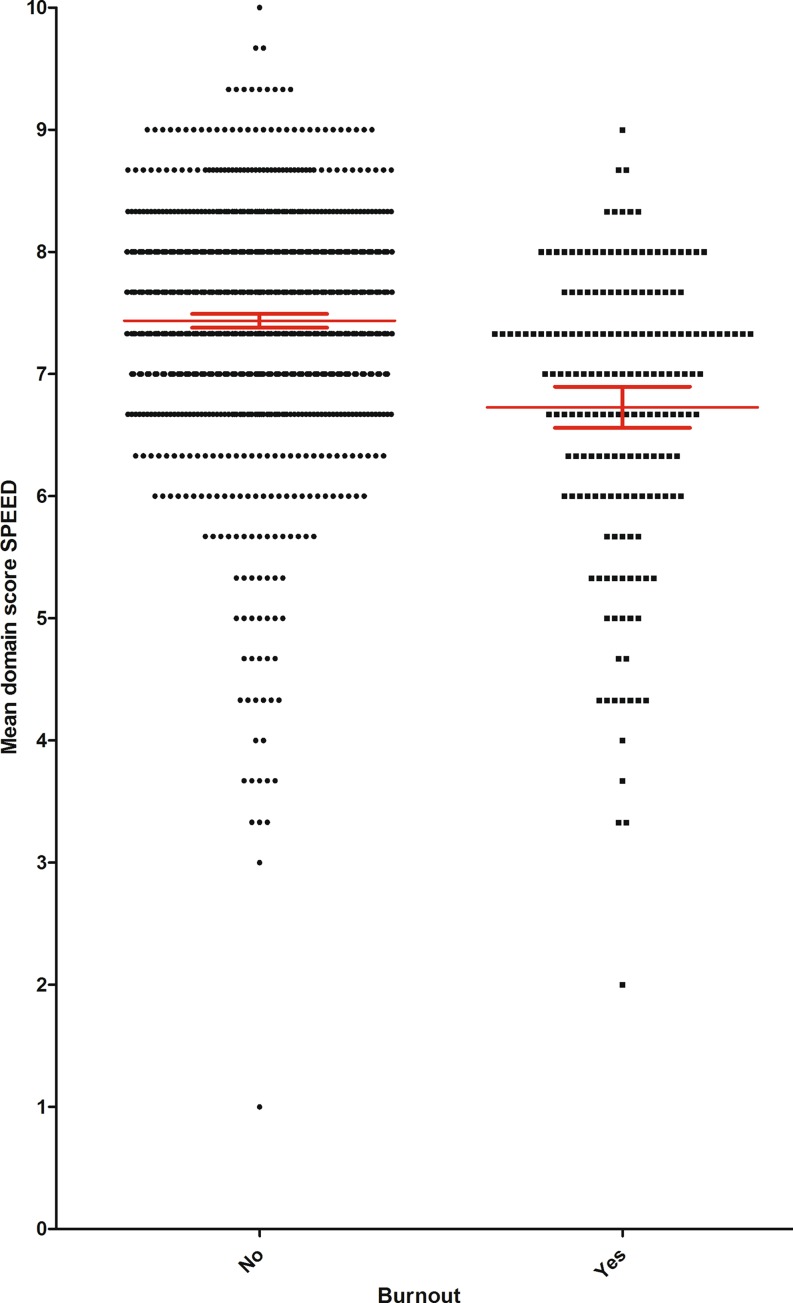
Mean SPEED (Scan of Postgraduate Educational Environment Domains) scores of residents with (*n* = 185) and without (*n* = 1,046) burnout. Horizontal bars represent mean values and T‑bars indicate 95% confidence intervals. The difference in SPEED score (0.71, 95% confidence interval for difference; 0.56–0.86) between both groups is significant (*p* < 0.001)

Concerning our primary research question, we found that residents without burnout gave significantly higher SPEED domain scores (mean, SD: 7.44, 0.94) than residents with burnout (mean, SD: 6.73, 1.16) (95% confidence interval for difference; 0.56 to 0.86, *p* < 0.001) (Fig. 1). The mean difference of 0.71 points in the perceived quality of the learning environment between residents with and without burnout is clinically relevant [15]. After adjustment for potential demographic and work-related predictors of burnout (Tab. 2), the association between SPEED score and resident burnout remained both relevant and statistically highly significant (aOR 0.54 for each point higher on the SPEED, 95% CI 0.46 to 0.62, *p* < 0.001). Univariate associations between the SPEED domain scores and the dimensions of burnout are displayed in Tab. 3. Moreover, we found a greater difference between the true number of hours spent working and the number of work-hours stated in the employment contract in residents with burnout (mean, SD: 9.49, 6.93) compared with those without burnout (mean, SD: 7.56, 6.26) (95% CI of difference 2.92 to 0.51, *p* < 0.001).

**Table 2:** Multivariate model: demographic and occupational factors independently associated with burnout in residents

	Odds ratio (95% CI)	*p*-value
Gender	0.94 (0.64–1.38)	0.76
Age	0.99 (0.95–1.04)	0.77
Marital status	1.15 (0.84–1.57)	0.38
Year of training	0.94 (0.83–1.06)	0.32
Teaching hospital	1.16 (0.97–1.39)	0.10
Type of specialty	0.66 (0.50–0.86)	0.002
SPEED score	0.54 (0.46–0.62)	<0.001

**Table 3:** Associations between the SPEED (Scan of Postgraduate Educational Environment Domains) scores (95% confidence interval of difference) and overall burnout and the three dimensions of burnout in Dutch residents from 33 specialties

		Mean score 3 items SPEED (SD)	95% CI of difference	*p*-value
Overall burnout	Burnout	6.73 (1.16)	0.56 to 0.86	<0.001
	No burnout	7.44 (0.94)		
Emotional exhaustion	Exhausted	6.87 (1.20)	0.49 to 0.74	<0.001
	Not exhausted	7.49 (0.88)		
Depersonalization	Depersonalization	6.99 (1.06)	0.32 to 0.58	<0.001
	No depersonalization	7.44 (0.97)		
Personal accomplishment	Not competent	6.85 (1.31)	0.71 to 0.39	<0.001
	Competent	7.40 (0.93)		

## Discussion

In a large national sample of Dutch residents from 33 different specialties we found that 15% met the criteria for moderate to severe burnout. Residents without burnout gave a higher rating for the quality of the learning environment than residents with burnout. We found a significant inverse association between the perceived quality of the learning environment and emotional exhaustion, depersonalization, and reduced personal accomplishment. The inverse association between the perceived quality of the learning environment and the risk of burnout remained highly significant after controlling for gender, age, marital status, year of training, teaching hospital and type of specialty.

Several recent studies have reported a high prevalence of burnout among residents [1, 3, 17]. Growing awareness of the detrimental consequences of burnout on patient care and the personal lives of residents raises the question which factors drive burnout. Individual factors such as personality traits [19] and demographics [3] probably influence the way stressors are perceived by residents and could therefore contribute to the development of burnout. However, the key contributor is believed to be the learning environment [6, 17]. The results of the present study confirm and extend our earlier observations of a highly significant inverse association between the quality of the learning environment and the risk of burnout among residents [17].

Several aspects of the learning environment have been implicated to play a role in the development of burnout: long working hours [20], lack of autonomy [21], and lack of reciprocity [22]. Important aspects of the learning environment include aspects of supervisory support, accessibility of supervisors, teamwork (peers, nurses and other hospital personnel) and mutually supportive and beneficial relationships with supervisors [23]. Interventions to improve the learning environment could focus on creating a safe atmosphere with sufficient autonomy for residents [21], with supervisors who provide timely and useful feedback [24] and are attentive to the educational needs of residents [25]. Recognition of the importance of the learning environment has led to quality-improvement initiatives such as the Clinical Learning Environment Review (CLER) program in the United States [26]. Specific guiding findings of the first CLER report include: improving patient safety by applying a system-based approach, improving engagement in interprofessional collaboration, achieving greater understanding in appropriate titration of supervision, and paying attention to workload and work conditions to address fatigue and burnout in residents [27].

Long work hours and high workload are associated with increased fatigue-related errors and a lower likelihood of participation in educational activities [28, 29]. This has led to reforms that have further reduced resident work hours in many countries. Although the true effect of the number of hours worked on the development of resident burnout remains controversial, a recent study indicates that a longer working week does increase the risk of burnout in residents [20]. In the present study, we found an association between burnout and the number of hours worked beyond their employment contract. Reducing the workload for residents, the frequency of on-call duty and increasing participation of supervisors is associated with higher residents’ satisfaction with the quality of the learning environment [30]. Based on these findings and our own observations, we speculate that improving the learning environment by reducing the workload is of particular importance in the prevention of resident burnout.

Our study is the first to describe a consistent association between the learning environment and burnout. Another strength of our study is the nationwide recruitment of residents from all specialties and teaching hospitals. In addition, we used the complete MBI to determine burnout whilst most previous studies on burnout in medical professions used abbreviated versions [31]. The ability to adjust for working hours and perceived work-life balance allowed a robust and consistent analysis between the quality of the learning environment and the risk of burnout. We acknowledge the following limitations. Firstly, we were not able to determine a reliable response rate. Selective non-participation of residents with burnout cannot be excluded, which would mean that the burnout rate we reported would be an underestimate of the true burnout risk among residents. This limitation, however, applies to all studies assessing burnout among residents, so the burnout rate we reported can be reliably compared with those obtained in earlier work. Although the response rate in our study was relatively low, the study sample was large enough to allow meaningful comparisons between subgroups. Our study sample was representative in terms of age and type of specialty of the root population of all Dutch residents. Although women were overrepresented in our study sample, burnout rates were comparable between female and male residents in our study, and we adjusted for gender in the multivariate analysis of the association between learning environment and burnout, which was the primary focus of our study. Secondly, the cross-sectional design of the study precluded causal inference.

## Conclusion

Despite the merits of becoming a medical specialist, residents are at risk of developing burnout. This has a tremendous impact not only on the personal lives of the residents but also on the quality of the patient care they deliver. Our study suggests that the quality of the learning environment as perceived by residents is a major determinant of the risk of resident burnout. Residents, supervisors, educators and policymakers have a shared responsibility to promote a rich learning environment in which residents flourish with minimum risk of burnout.
